# Tailoring Plasmonic Nanoheaters Size for Enhanced Theranostic Agent Performance

**DOI:** 10.3390/bioengineering11090934

**Published:** 2024-09-18

**Authors:** Túlio de L. Pedrosa, Gabrielli M. F. de Oliveira, Arthur C. M. V. Pereira, Mariana J. B. da S. Crispim, Luzia A. da Silva, Marcilene S. da Silva, Ivone A. de Souza, Ana M. M. de A. Melo, Anderson S. L. Gomes, Renato E. de Araujo

**Affiliations:** 1 Laboratory of Biomedical Optics and Imaging, Federal University of Pernambuco, Recife 50740-540, Brazil; tulio.pedrosa@ufpe.br (T.d.L.P.); gabrielli.oliveira@ufpe.br (G.M.F.d.O.); mariana.crispim@ufpe.br (M.J.B.d.S.C.); 2 Department of Physics, Federal University of Pernambuco, Recife 50670-901, Brazil; arthur.messias@ufpe.br (A.C.M.V.P.); anderson.lgomes@ufpe.br (A.S.L.G.); 3 Graduate Program in Biological Sciences, Federal University of Pernambuco, Recife 50670-420, Brazil; luzia.abilio@ufpe.br; 4 Laboratory of Pharmacology and Experimental Cancerology, Federal University of Pernambuco, Recife 50740-521, Brazil; marcilene.souza1989@gmail.com (M.S.d.S.); ivone.souza@ufpe.br (I.A.d.S.); 5 Department of Biophysics and Radiobiology, Federal University of Pernambuco, Recife 50670-901, Brazil; ana.mamelo@ufpe.br

**Keywords:** gold nanoparticles, photoacoustic imaging, photothermal therapy, size optimization, S180 cells

## Abstract

The introduction of optimized nanoheaters, which function as theranostic agents integrating both diagnostic and therapeutic processes, holds significant promise in the medical field. Therefore, developing strategies for selecting and utilizing optimized plasmonic nanoheaters is crucial for the effective use of nanostructured biomedical agents. This work elucidates the use of the Joule number ($J_{o}$) as a figure of merit to identify high-performance plasmonic theranostic agents. A framework for optimizing metallic nanoparticles for heat generation was established, uncovering the size dependence of plasmonic nanoparticles optical heating. Gold nanospheres (AuNSs) with a diameter of 50 nm and gold nanorods (AuNRs) with dimensions of 41×10 nm were identified as effective nanoheaters for visible (530 nm) and infrared (808 nm) excitation. Notably, AuNRs achieve higher $J_{o}$ values than AuNSs, even when accounting for the possible orientations of the nanorods. Theoretical results estimate that 41×10 nm gold nanorods have an average Joule number of 80, which is significantly higher compared to larger rods. The photothermal performance of optimal and suboptimal nanostructures was evaluated using photoacoustic imaging and photothermal therapy procedures. The photoacoustic images indicate that, despite having larger absorption cross-sections, the large nanoparticle volume of bigger particles leads to less efficient conversion of light into heat, which suggests that the use of optimized nanoparticles promotes higher contrast, benefiting photoacoustic-based procedures in diagnostic applications. The photothermal therapy procedure was performed on S180-bearing mice inoculated with 41×10 nm and 90×25 nm PEGylated AuNRs. Five minutes of laser irradiation of tumor tissue with 41×10 nm produced an approximately 9.5% greater temperature rise than using 90×25 AuNRs in the therapy trials. Optimizing metallic nanoparticles for heat generation may reduce the concentration of the nanoheaters used or decrease the light fluence for bioscience applications, paving the way for the development of more economical theranostic agents.

## 1. Introduction

Metallic nanostructures have gained significant attention over the past decade as important materials for optical applications, including medical diagnostics and optical therapies. Metallic nanoparticles (NPs) in various shapes exhibit excellent optical absorption in the visible and near-infrared (NIR) regions, showing substantial potential for applications requiring photothermal conversion. The large optical absorption of metallic nanostructures arises from the collective oscillation of the metal conduction electrons, a phenomenon known as localized surface plasmon resonance (LSPR). In numerous applications where plasmonic NPs are utilized, managing heat is often a significant challenge, and temperature increase becomes a major problem to overcome. For instance, local heating caused by plasmonic structures can induce changes in the surrounding refractive index due to the thermo-optic effect [1] or can lead to the denaturation of biomolecules and proteins in biosensing contexts [2].

There are medical procedures where the generation of heat is beneficial. In this context, the LSPR phenomenon has been harnessed in photothermal processes for the destruction of cells and tissues, including microorganisms and malignant tumors [3,4], by exploiting the electric field damping via the Joule effect. In thermal treatments, well-localized heating is desired to increase the temperature in a restricted tissue volume. Thermal therapy depends not only on the final temperature achieved but also on the duration for which the heating is sustained. Specifically, photothermal therapy (PTT) relies on temperature increases due to light (visible and infrared) absorption for the localized thermal destruction of biological structures [5]. In particular, the use of infrared (IR) light sources within the biological window enables deep tissue (a few centimeters) intervention. Within this biological window, absorption and scattering processes are minimized, resulting in partial transparency of tissues. Moreover, two spectral bands are considered ideal for optical therapy: (i) the first biological window, ranging from 700 nm to 980 nm; and (ii) the second biological window, extending from 1000 nm to 1400 nm [6].

Processes and inputs that enhance the efficiency of thermal therapies are targets of scientific and medical attention. Gold nanoparticles, such as nanospheres [7,8], nanoshells [9], nanorods [10,11,12,13,14], nanocubes [15,16], nanohexapods [11], and nanourchins [17], have been evaluated in numerous photothermal cancer treatments. For example, thermal treatments for oral [12] and colon [7,13] carcinomas in mice have been assessed, and gold nanocages have been effectively used for the selective photothermal destruction of breast cancer both in vitro [15] and in vivo [16].

A key consideration in PTT is the effective incorporation of nanoheaters into the target tissues or cells. NP targeting of cancer cells can be passive, involving direct intratumoral administration, or active, involving functionalization that enhances NP binding to tumor tissues following intravenous administration [18]. For instance, red blood cell membranes embedded with NIR dye and camouflaging Mn-ferrite NPs have been used as theranostic nanocarriers to induce immunogenic cell death in the treatment of S180-bearing mice [19]. Murine Sarcoma 180 (S180) is a cell line originating from murine mice. S180 cells grow rapidly, with tumors reaching approximately 1 cm^3^ within seven days of subcutaneous implantation [20]. These cells are commonly used in experimental pre-clinical cancer studies to understand tumor growth mechanisms, test new therapies, and evaluate the effectiveness of various therapeutic agents [21,22].

Metallic nanoparticles have been extensively studied for their potential in photoacoustic imaging (PAI) [23]. PAI merges the high resolution of optical imaging with the deep tissue penetration capabilities of ultrasound [24]. In PA imaging techniques, such as tomography, microscopy, and endoscopy, laser light is directed at the target tissue, converting part of the light energy into heat. This heat induces transient thermoelastic expansion, generating an acoustic signal. Consequently, the amplitude of PA signals is correlated with temperature increases and is inherently linked to photothermal effects [25]. Detecting these optically generated ultrasonic waves allows for the construction of detailed 2D and 3D images, enabling tissue mapping. For cancer treatment, PAI is an effective tool for the prediction of tumor recurrence and as a way to probe the therapeutic efficacy of photodynamic and photothermal therapies [26,27,28].

There are two primary modalities of PAI: photoacoustic microscopy (PAM) and photoacoustic tomography (PAT). Each offers a balance between optical and acoustic resolution. In PAM, a focused laser beam achieves high spatial resolution with limited penetration depth, while PAT uses diffuse illumination for deeper penetration, with spatial resolution determined by the ultrasonic transducer array and reconstruction algorithms [29,30]. A single ultrasonic detector in PAT provides lower spatial resolution, while the use of detector arrays is capable of enhancing spatial resolution to levels comparable with PAM systems [24,30,31].

Various endogenous chromophores that absorb light, such as oxyhemoglobin and deoxyhemoglobin, have been utilized as PAI contrast agents for visualizing physiological processes. These markers are crucial for applications like noninvasive analyses of human palmar vessels [32], human brain imaging [33], neurological studies [34], and identifying breast cancer tumors [35]. To overcome PAI limitations, various exogenous contrast-enhancing nanoagents have been investigated for use in PAI [36,37,38,39,40]. In particular, the use of plasmonic nanoheaters as contrast agents offers advantages such as high selectivity through wavelength tuning and targeting functionalization. Additionally, the high temperatures achieved by nanostructures supporting plasmon resonance significantly enhance visibility during PAI [41].

Mantri and Jokerst proposed using the absorption coefficient of plasmonic colloids as a key parameter in selecting plasmonic contrast agents for PAI [42]. However, this approach is limited, as it conflates the collective and individual contributions of the nanoheaters, hindering the identification of optimized contrast nanoagents for PAI.

Notably, the introduction of optimized nanoheaters (theranostic agents) that allow the integration of diagnostic and therapeutic processes is of great interest in the medical field [11]. Therefore, identifying strategies for the selection and use of optimized plasmonic nanoheaters contributes to the rational use of nanostructured biomedical agents. This work discusses and demonstrates the use of figures of merit in identifying high-performance plasmonic nanoheaters. Optimized gold nanospheres and nanorods are established as theranostic agents for in vivo photothermal cancer therapy and photoacoustic imaging. Moreover, our approach provides insights for better exploiting localized surface plasmon resonance in optical imaging and therapy.

### Plasmonic Heating

The light interaction with a metallic nanoparticle can be quantified by the absorption ($\sigma_{abs}$) and scattering ($\sigma_{sca}$) cross sections, which are highly reliant on particle size, shape, and material. Based on the quasi-static approach, the optical cross sections for spherical NPs can be written as [43]:

(1) $$\sigma_{sca}=\frac{8\pi k^{4}r^{6}}{3}\frac{\left(\epsilon^{\prime }-\epsilon_{m}\right)+\epsilon^{\prime \prime 2}}{{\left(\epsilon^{\prime }+2\epsilon_{m}\right)}^{2}+\epsilon^{\prime \prime 2}}\;\;\mathrm{and}$$

(2) $$\sigma_{abs}=4\pi kr^{3}\frac{\epsilon_{m}\epsilon^{\prime \prime }}{{\left(\epsilon^{\prime }+2\epsilon_{m}\right)}^{2}+\epsilon^{\prime \prime 2}}\;,$$

in which *k* is the wavenumber of incident light, $\epsilon^{\prime }$ and $\epsilon^{\prime \prime }$ are the real and imaginary parts of the NP complex permittivity ($\epsilon =\epsilon^{\prime }+j\epsilon^{\prime \prime }$), $\epsilon_{m}$ is the medium permittivity, and *r* is the nanosphere radius. Equations (1) and (2) indicate that all cross sections increase considerably and peak when the denominator is minimized This condition states that the real part of the nanosphere permittivity must be negative $\epsilon^{\prime }=-2\epsilon_{m}$ at the same time that $\epsilon^{\prime \prime }$ must be small, a constraint known as the Fröhlich condition. If the Fröhlich condition is met, the forced oscillation of the conduction electrons is sustained, giving rise to the localized surface plasmon resonance [43]. Equations (1) and (2) also show that the cross sections of nanoparticles increase with their size; in other words, the larger the nanoparticle, the greater the amount of energy absorbed or scattered by the structure.

The spectral region in which LSPR occurs depends on the free carrier density of the NP material [43]. Noble metals such as Cu, Ag, and Au constitute the most commonly studied plasmonic materials, as their high density of carriers place the LSPR in the visible region, while non-noble metals, such as Pb and Al, exhibit LSPR frequencies in the UV region of the spectrum and display broader LSPR bands [44,45].

As the size of an NP increases, the non-homogeneity of the electric field in the NP induces dephasing to the oscillating electrons and creates retardation effects, inducing the broadening and red shift of plasmon peak [44]. In most cases, new resonant modes arise due to anisotropy in NP shape, which is especially true for gold nanorods (AuNRs). For AuNRs, size is directly related to longitudinal mode LSPR tunability [46] and tied to its aspect ratio (AR), which is defined as the ratio of its length (L) to its width (D). This tunability enables near-infrared operation AuNRs and provides higher absorption structures, which makes them exceptionally desirable for biomedical applications [47]. Although heat generation within the nanostructure undergoes significant spatial variation, the temperature of the nanoparticle remains nearly uniform due to rapid thermal diffusion and its small size [48].

On selecting metallic nanoparticles for thermal applications, structures with a high absorption cross section are desired. As the particle size increases, both absorption and scattering rise, with scattering becoming the dominant interaction. A higher absorption cross section does not necessarily translate to an increase in temperature or heat delivery. The ability of a particle to increase its temperature upon light absorption and to dissipate heat to its surroundings is related to its surface area and volume. For efficient heat dissipation, the volume should be minimized, while the surface area should be maximized. This is where particle morphology becomes crucial in photothermal processes.

In nanoparticle heating by pulses with time width in the order of the NP’s thermalization time, all the energy absorbed by the plasmonic nanoparticle will be employed to elevate its temperature as long as no phase transitions are involved. Therefore, temperature variation in the nanoparticle ($\Delta T_{np}$) is given by:

(3) $$\Delta T_{np}=\frac{1}{\rho C_{p}}\int_{0}^{t}\left(\frac{\sigma_{abs}}{V_{np}}\right)I\left(t^{\prime }\right)dt^{\prime }\;,$$

in which ρ is the NP density, $V_{np}$ is the NP volume, and $C_{p}$ is the specific heat of the NP. The NP temperature variation is directly proportional to the ratio of its absorption cross section to volume. Thus, NP morphology becomes relevant in thermoplasmonic applications. An effective way to evaluate the ability of an NP to generate heat is by exploring the Joule number ($J_{o}$), given by [49]:

(4) $$J_{o}=\frac{\lambda_{ref}}{2\pi }\frac{\sigma_{abs}}{V_{np}}\;,$$

where $\lambda_{ref}\approx 1240$ nm is the reference wavelength of a photon with energy of 1 eV. Hence, $J_{o}$ arises as the appropriate figure of merit (FoM) to assess temperature variation in single NPs and colloidal suspensions induced by laser excitation [50].

In colloidal heating, the overall global temperature change is achieved by the superposition of each individual nanoparticle’s contribution. In this scenario, each nanoparticle can be considered a point heat source. Consequently, the overall global temperature is proportional to the temperature of each plasmonic nanoheater.

The outcome of a photothermal intervention relies on the light intensity used and the laser’s incident time, as described in Equation (3). Specifically, for pulsed lasers, the source repetition rate and duty cycle are also crucial in determining the thermal load on the sample [51], which in turn dictates the outcome of the thermal optical treatment. Controlling these factors is crucial not only for establishing a hyperthermia process but also for inducing coagulation or even ablation of the target tissue.

## 2. Materials and Methods

### 2.1. Computational Analysis

**Figure 1:**
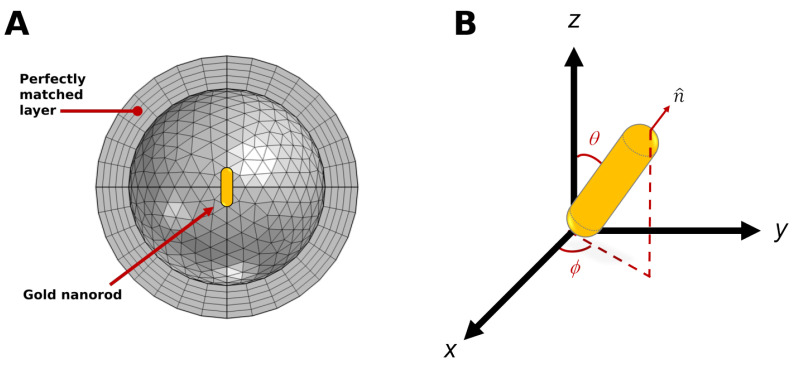
(**A**) Meshed simulation setup used for nanorod optimization. (**B**) Gold nanorod geometry and spatial coordinates of orientation. The electric field of incident light is polarized along the *x*-axis, and the direction of the light propagation is along the *z*-axis.

The absorption cross sections of gold nanospheres were obtained using Mie theory. The nanosphere diameter was varied from 5 to 100 nm for $J_{o}$ value analysis. For nanorods, plasmonic properties were appraised by means of finite element method (FEM) electromagnetic simulations in COMSOL Multiphysics, where a single AuNR of length L and diameter D was placed in a dielectric medium (water). The surrounding medium refractive index was assumed to be wavelength-independent and bounded by a perfectly matched layer (PML) with spherical symmetry, mimicking an open boundary and avoiding the reflection of scattered light. AuNR length (L) and diameter (D) were swept from 15 nm up to 200 nm. Figure 1A illustrates the cross section of the meshed model. Due to the elongated shape of the AuNRs, their relative orientation to the polarization of incident light is significant to the nanostructure absorption cross section value and, consequently, affects the optical heating of plasmonic nanostructures [52]. Figure 1B shows the possible orientations of AuNRs, considering an incident electric field with polarization fixed along the *x*-axis. In Figure 1B, the unit vector $\hat{n}$ is parallel to the long axis of AuNR. The angle ϕ is in the *x*–*y* plane, which is obtained from the *x*-axis to the projection of unit vector $\hat{n}$ on the *x*–*y* plane, where the angle between $\hat{n}$ and the *x*–*y* plane is θ. Here, averaged $J_{o}$ values (${\langle J_{o}\rangle }_{\theta ,\phi }$) were obtained considering θ and ϕ values ranging from 0 to 90°.

The bulk permittivity ($\epsilon_{bulk}\left(\omega \right)$) values of gold were obtained from Johnson and Christy’s reported results [53]. However, as the size of the metallic structure decreases, the probability of electron NP surface scattering increases. This is specially significant when the NP size has dimensions comparable to the mean free path of the conduction electrons. This aspect leads to a reduction in the electrons’ mean free path, causing the permittivity of metallic NPs to be size-dependent [45]. Therefore, the size dependence of NP permittivity is given by [54]:

(5) $$\epsilon \left(\omega \right)=\epsilon_{bulk}\left(\omega \right)+\frac{\omega_{p}^{2}}{\omega^{2}+j\omega \gamma_{0}}-\frac{\omega_{p}^{2}}{\omega^{2}+j\omega \left(\gamma_{0}+A\frac{v_{F}}{L_{eff}}\right)}\;,$$

where $L_{eff}$ is the effective mean free path given by $L_{eff}=4V_{np}/S$ for convex shapes. Here, *S* is the NP surface area. The surface scattering constant (*A*) is an empirical parameter that describes the scattering at the surface of nanostructures. For spheres, *A* is usually considered to be equal to 1 [54], while the surface scattering parameter for AuNRs lies between 0.25 and 0.5 [55,56]. Moreover, $\omega_{p}$ is the plasma frequency ($1.369\times 10^{16}$ rad/s) [57], and $\gamma_{0}$ is the bulk damping parameter ($1.07\times 10^{14}$ s^−1^) [57].

To validate the computational procedure, its results were thoroughly evaluated by comparing them with spectroscopic results reported in the existing literature [50,58].

### 2.2. Theranostic Photothermal Nanoagents

#### 2.2.1. Gold Nanoparticle Samples for Photoacoustic Analyses

Based on computational results, AuNS samples were selected to evaluate the size-dependent PA signal generation of gold colloids at the 530 nm wavelength. Citrate-stabilized colloidal samples of monodispersed AuNSs in deionized water (less than 12% variability in size and shape), with diameters of 5, 50, and 100 nm, were acquired from Sigma-Aldrich (St. Louis, MO, USA). Likewise, AuNRs of three different sizes were chosen to assess PA signal generation of colloidal gold under 808 nm laser irradiation. CTAB-stabilized colloidal samples of AuNRs in deionized water with sizes 41×10 nm, 90×25 nm, and 134×40 nm were acquired from Nanopartz, Inc. (Loveland, CO, USA).

All samples were diluted in distilled water, leading to solutions of the same total mass of gold. For AuNSs, the final concentration among samples was 17.4 μg/mL, while for AuNRs, it was 42 μg/mL. Samples were analyzed by UV-Vis spectroscopy (Ocean Optics USB2000) and scanning electron microscopy (SEM) before and after the experiment to ensure that the NPs were not degraded.

#### 2.2.2. Gold Nanoparticle Samples for PTT Analyses

AuNR of sizes 41×10 nm and 90×25 nm were chosen to experimentally evaluate the collective heating of colloidal samples under laser irradiation. Both AuNR sizes have “matching spectra”, exhibiting plasmonic peaks at the same wavelength. Colloidal samples of polyethylene glycol-coated (PEGylated) AuNRs suspended in PBS were acquired from Nanopartz, Inc. (Loveland, CO, USA). The stock concentrations of the acquired AuNR samples were 2.5 mg/mL and 4 mg/mL for sizes 41×10 nm and 90×25 nm, respectively.

### 2.3. Photoacoustic Microscopy (PAM)

**Figure 2:**
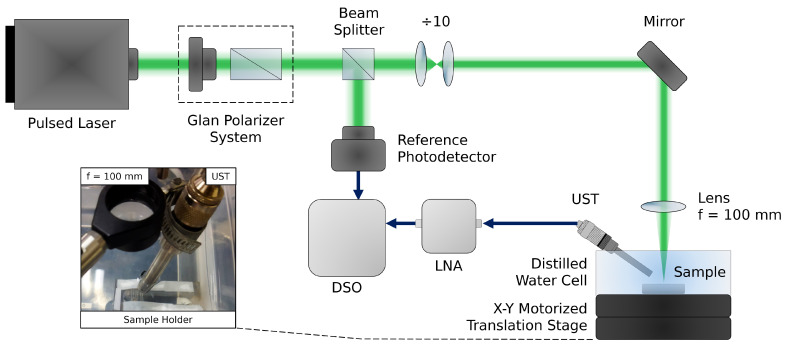
Schematic details of the PAM setup. The beam fluence is controlled by a Glan polarizer system, and a small part of the beam is directed to a reference photodetector to trigger the digital storage oscilloscope (DSO) for data acquisition. The beam diameter is reduced 10× and focused on the sample through a 100 mm convex lens. The sample is swept by the X–Y motorized translation stage, and the PA signal acquired through the ultrasonic transducer (UST) is conditioned by the low-noise amplifier (LNA) prior to being recorded by the DSO. The inset features the distilled water cell, showing the focusing lens and UST positioning, as well as the sample holder.

PA characterization was carried out by employing a customized PAM setup shown in Figure 2 and previously described in Das et al. [59]. In short, laser light from an optical parametric oscillator (OPO) (OPOTEK, Carlsbad, CA, USA, Vibrant 355 LD, 0.4–2.4 µm, 5 ns) operating at 10 Hz was focused on the samples, consisting of three medical polyvinyl chloride (PVC) capillary tubes (from an IV infusion set) positioned side-by-side. The tubes, of acoustic impedance of 3.2 × 10^6^ Rayl [60], have an internal (external) diameter of 1.25 mm (2 mm) and were cleaned via multiple alcohol rinses prior to sample injection. Gold NP samples were sealed inside the capillary tubes with thermoplastic adhesive and attached to an acrylic holder to provide mechanical stability for the measurements. Two holders with three capillary tubes each were prepared, one for the AuNSs (5, 50, and 100 nm) and one for the AuNRs (41×10, 90×25, and 134×40 nm). Subsequently, each acrylic holder was submerged into the distilled water cell for characterization, as displayed in the inset of Figure 2. AuNS and AuNR samples were irradiated by 530 and 808 nm, respectively, and laser fluence was limited to 20 mJ/cm^2^ to comply with the exposure limit of pulsed nanosecond lasers for bioapplications (514 nm) [61].

The water immersion ultrasonic transducer (UST) (Olympus V310-N-SU, 5 MHz) was positioned at an angle to maximize the PA signal from the sample and minimize the effects of reflection and absorption at other interfaces, allowing the focused beam (f = 100 mm) to reach the sample. The sample was scanned using an X–Y motorized translation stage placed under the water cell, comprised of two 50 mm linear stages (MTS50-Z8—50 mm, Thorlabs, Inc., Newton, NJ, USA). The acquired signal was amplified 19.4 times using a radio frequency low-noise amplifier (LNA) (ZFL-500LN-BNC) connected to a digital storage oscilloscope (DSO).

### 2.4. Photothermal Therapy

#### 2.4.1. Tumor Implantation

For in vivo assays, male albino Swiss mice (*Mus musculus*) weighing 25–35 g were used. The mice were housed and kept in polypropylene cages with free access to food and water in a room with total air renewal 15 times per hour, under controlled lighting conditions (12 h light/dark cycle) at 22 ± 2 °C and humidity between 55 and 65%. The handling of the animals during the experiment was approved by the Comitê de Ética em Experimentação Animal (Ethics Committee for Animal Experimentation) of Federal University of Pernambuco (UFPE), under process #0088/2023 and in accordance with the National Institute of Health’s “Guide for the Care and Use of Laboratory Animals” [62]. S180 tumor cells were obtained from UFPE’s Department of Antibiotics (Recife, Pernambuco, Brazil) and were maintained in mice through weekly intraperitoneal injections. Ascites withing S180 tumor cells germinated for 7 days, after which it was aspirated and centrifuged (70 g, 5 min, 4 °C). Cell counting and cell viability testing with Trypan Blue were performed using the sediment, and the concentration of viable cells was adjusted with sterile 150 mM NaCl solution to $5.0\times 10^{6}$ cells/mL. After preparation, the ascites tumor was implanted subcutaneously in the right axillary region of experimental mice (0.1 mL) for growth in solid form. Tumor progression was assessed until the establishment of a palpable tumor mass with diameter of approximately 1.0–1.5 cm and with an effective surface area of approximately 0.78 cm^2^.

#### 2.4.2. PTT Laser Heating

The in vivo PTT procedure took place after 7–10 days of tumor growth. Then, the S180-bearing mice were subjected to laser irradiation as well as injection of nanoparticles and saline (control). Animals were anesthetized with 2% xylazine (10 mg/kg) and 10% ketamine hydrochloride (75 mg/kg). The tumor region was shaved and the mice placed in a supine position on the irradiation bed. Subsequently, 200 μL of an aqueous suspension containing PEGylated AuNRs (100 μg/mL) or saline (0.9%) were injected intratumorally immediately before irradiation. Two distinct AuNR sizes were tested (41×10 and 90×25 nm). The animals were continuously irradiated by a pulsed laser (Chameleon Vision II, 140 fs/80 MHz; Coherent, Santa Clara, CA, USA) at 808 nm. The laser power was adjusted to provide irradiance equivalent to 1.0 W/cm^2^ (12.5 nJ/cm^2^), in accordance with the maximum permissible exposure (MPE), and a total dose of 600 J/cm^2^ was delivered to all rodents for 10 min of irradiation, during which thermographs were acquired in real time using a thermal imaging camera (Flir E4; Flir, Wilsonville, OR, USA). Throughout the irradiation process, a physical barrier was used to protect the animal’s face from diffuse reflections and to prevent blindness. Mice were sacrificed 72 h after irradiation, and tissue histology was performed on the irradiated tumors.

## 3. Results

**Figure 3:**
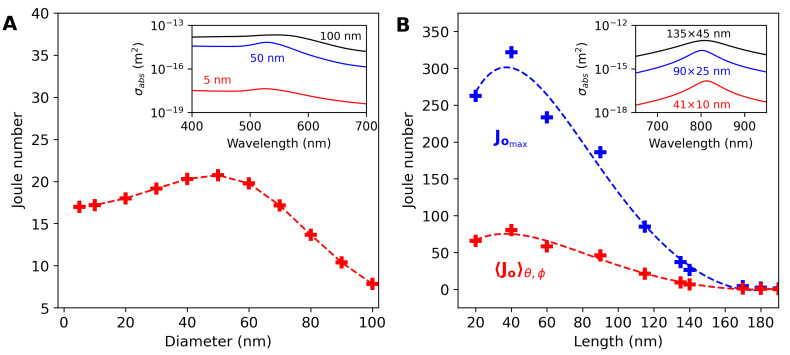
Joule number values of (**A**) NSs and (**B**) NRs as function of NP size. The insets show the ($\sigma_{abs}$) spectrum of the nanostructures in water.

Figure 3A depicts Joule number values for gold nanospheres of different sizes (diameters), considering their maximum absorption cross sections at the plasmonic peak in water. The inset in Figure 3A shows the absorption cross section spectrum of nanospheres with diameters of 5, 50, and 100 nm, indicating that, as the particles increase in size, their plasmonic peak remains at approximately 530 nm (with a small red shift), but their cross section values significantly increase (more than a thousandfold). However, the nanosphere $J_{o}$ value is higher for smaller particles. The gold nanosphere with a 50 nm diameter shows $J_{o}$ values up to 20.2.

Figure 3B depicts the maximum theoretical Joule number values ($J_{o\;max}$, blue cross) for AuNRs as function of NP length. The maximum $J_{o}$ values are achieved considering the NP long axis parallel to the polarization of light. The average $J_{o}$ values (${\langle J_{o}\rangle }_{\theta ,\phi }$, red cross), considering θ and ϕ values ranging from 0 to 90°, are displayed in Figure 3B. The inset of Figure 3B depicts the absorption cross section spectrum for AuNRs of sizes 41×10, 90×25, and 135×45 nm, indicating an AuNR plasmonic peaks close to 808 nm. The dashed lines in Figure 3 serve as a visual guide for the points (+) obtained from the theoretical models.

**Figure 4:**
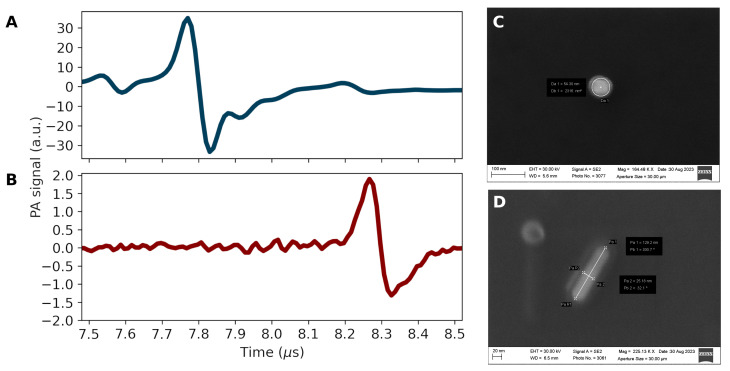
(**A**) Photoacoustic signal from 50 nm AuNSs (solid blue line, λ = 530 nm). (**B**) Photoacoustic signal from 90×25 nm AuNRs (solid red line, λ = 808 nm). (**C**) SEM of 50 nm AuNSs after photoacoustic measurement. (**D**) SEM of 90×25 nm AuNRs after photoacoustic measurement.

Metallic nanoparticles can be used as contrast agents for photoacoustic imaging. Figure 4A,B illustrate the photoacoustic response of samples containing 50 nm AuNSs (solid blue line, λ=530 nm) and 90×25 nm AuNRs (solid red line, λ=808 nm), respectively, after one laser pulse. SEM for the 50 nm AuNS and 90×25 nm AuNR samples taken after photoacoustic measurement are shown in Figure 4C,D, respectively.

**Figure 5:**
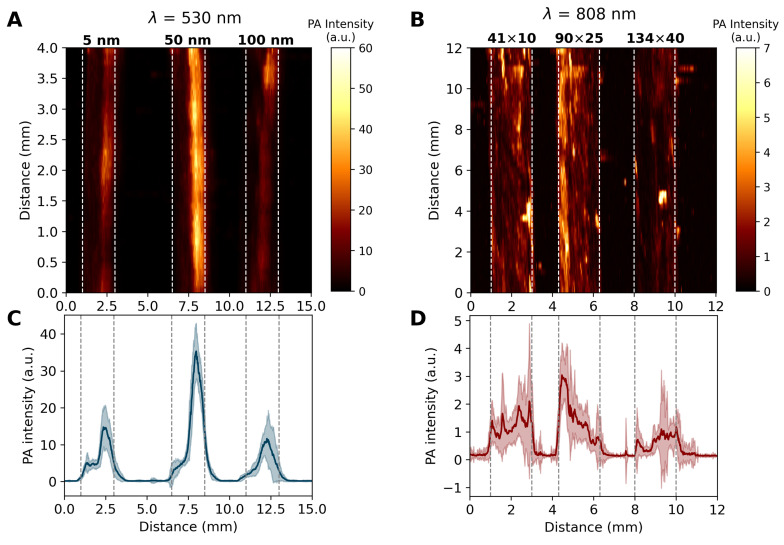
Experimental PAMs and PA intensities of colloidal gold samples containing NPs of different sizes. (**A**) PAM of 5, 50, and 100 nm AuNS samples (λ = 530 nm, FoV = 15×4 mm). (**B**) PAM of 41×10, 90×25, and 134×40 nm AuNS samples (λ = 808 nm, FoV = 12×4 mm). The color bar depicts the PA intensities of the PAMs (virtual colors), and the vertical dashed lines indicate the boundary of the capillary tubes. (**C**) Average PA intensity profile of the AuNS samples along the tubes (solid blue line) ± error range (light blue filled region). (**D**) Average PA intensity profile of the AuNR samples along the tubes (solid red line) ± error range (light red filled region).

To attest the NP size dependence effect on the PA signal generation, the produced intensities were recorded for the prepared AuNS and AuNR samples. Figure 5A depicts the PAM image for the AuNS sample (λ=530 nm), covering a field of view (FoV) of 15×4 mm, whereas Figure 5B (λ=808 nm) shows the PAM image obtained for the AuNR sample, covering an FoV of 12×4 mm. In both cases, the capillary tubes containing the colloids were positioned on the acrylic base in ascending order of NP size, from left to right. The PA intensity behavior of the samples is denoted by the virtual color bar on the right-hand side of Figure 5A,B. The vertical dashed lines indicate the capillary tube’s lateral boundary, with a dimension of ∼2 mm. In Figure 5C, the average PA intensity profile of the AuNS samples (5, 50, and 100 nm diameter) along the tubes is represented by a solid blue line, with its error range represented by the light blue filled region. Similarly, in Figure 5D, the AuNR average PA intensity profile of 41×10, 90×25, and 134×40 nm for the AuNRs samples along the tubes is represented by the solid red line and the error range by the light red filled region.

**Figure 6:**
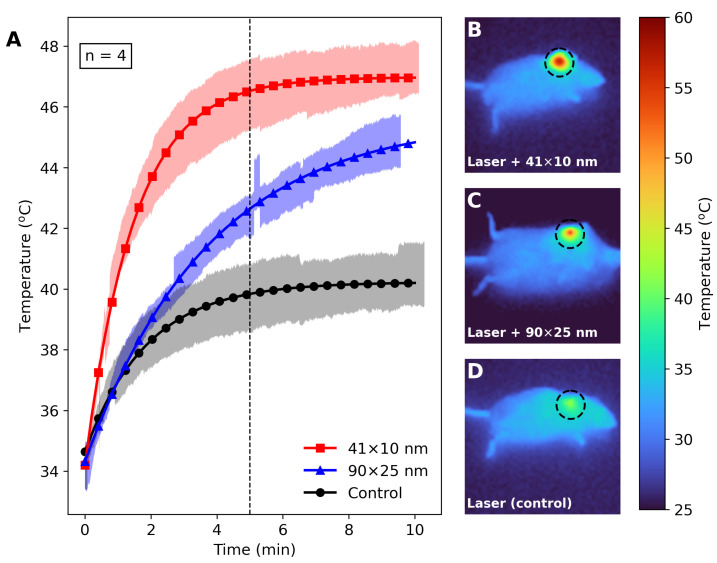
Photothermal performance on S180-bearing mice inoculated with AuNRs of different sizes and saline. (**A**) Average temperature of the irradiated tumors with error estimates (n = 4). The red line is the average temperature for 41×10 nm AuNRs, while the blue line shows the average temperature for 90×25 nm AuNRs. The black line describes the average temperature for saline. The color maps depict the thermographic stills at 10 min for the mice inoculated with (**B**) 41×10 nm AuNRs, (**C**) 90×25 nm AuNRs, and (**D**) saline.

The 41×10 nm and 90×25 nm PEGylated AuNRs were also evaluated as photothermal agents for PTT of S180-bearing mice. Figure 6A shows the average temperature of the irradiated tumors, inoculated with 41×10 nm AuNRs (red squares), 90×25 nm AuNRs (blue triangles), and saline (black circles). Figure 6B–D portray laser heating for mice with 41×10 nm AuNRs, 90×25 nm AuNRs, and saline, respectively. The red line in Figure 6A is the average temperature for tumors inoculated with AuNRs of size 41×10 nm (n=4), while the blue line describes the average temperature for tumors inoculated with AuNRs of size 90×25 nm (n=4). The black line describes the average temperature for tumors inoculated with saline (n=4). All average temperatures in Figure 6A were measured considering the thermal distribution inside the dashed lines of Figure 6B–D.

**Figure 7:**
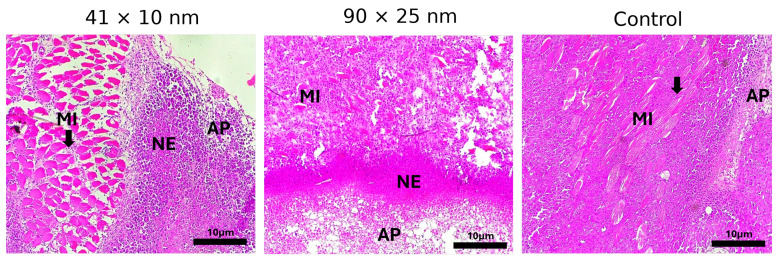
Photomicrographs of histological sections of surgical specimens of S180 implanted in rodents. (MI) Muscle invagination (black arrows), (AP) Apoptosis, and (NE) Necrosis. Scale bar: 10 μm.

Representative images of histological sections of surgical specimens of S180 tumors implanted in rodents are shown in Figure 7. Photomicrographs of histological sections stained with HE from the S180-bearing mice irradiated by a laser and injected with 41×10 nm PEGylated AuNRs, 90×25 nm PEGylated AuNRs, and saline (classified as the control group), are shown.

## 4. Discussion

The Joule number is a suitable figure of merit for identifying the photoheating performance of plasmonic nanoparticles. The NP $J_{o}$ value depends on the ratio of the absorption cross section to its volume. Figure 3A shows that the $J_{o}$ value for nanospheres with a diameter of 50 nm is approximately four times higher than that of the 100 nm nanoparticles, despite $\sigma_{abs}$ being greater for particles with larger diameters. de Pedrosa et al. experimentally demonstrated, using thermal lens spectroscopy, that particles with diameters of 5 and 50 nm reach higher temperatures when excited by a CW laser (532 nm) compared to particles with a diameter of 100 nm, following the trend indicated by the curve in Figure 3A [50]. However, only a small temperature difference was observed between the 5 and 50 nm nanospheres [50].

Figure 3B shows the maximum $J_{o}$ values and the absorption cross section spectrum for various AuNR sizes. For nanorods with plasmonic peak at 808 nm, the highest $J_{o}$ value was found for the structure with dimensions of 41×10 nm, even though the 90×25 nm and 135×45 nm nanorods have a $\sigma_{abs}$ that is seven times greater than that of the smallest structure. The maximum $J_{o}$ values shown in Figure 3B are slightly different than those reported in ref. [50], where Drude correction was not included in the calculation of nanostructure susceptibilities. When considering the photoheating of colloidal nanorods, the orientation of the nanostructure relative to the polarization of the incident light is crucial. For the 41×10 nm nanorod, the value of ${\langle J_{o}\rangle }_{\theta ,\phi }$ (80) corresponds to ∼25% of the $J_{o\;max}$ value (321). Additionally, for particles of 90×25 nm and 135×45 nm, the values found for ${\langle J_{o}\rangle }_{\theta ,\phi }$ are 46 and 9, respectively. Furthermore, the ${\langle J_{o}\rangle }_{\theta ,\phi }$ value for the 41×10 nm nanorod is four times greater than the $J_{o\;max}$ value of the 50 nm nanosphere, indicating that this nanorod is an efficient nanoheater for infrared photoheating.

In gold NPs, the energy absorbed from a short laser pulse is converted into heat in a matter of tens of picoseconds [63], which causes it to quickly heat up while still absorbing the nanosecond laser pulse. Also, the thermal relaxation time of a gold NP is considerably longer than the time it takes to heat up. For instance, for heat to diffuse 100 nm (in water), it takes about 100 ns [64], facilitating the NP localized thermal buildup that promotes its thermoelastic expansion and gives rise to the PA signal. Despite the local amplitude enhancement assisted by individual NPs, the resulting contrast emerges from the contribution of all NPs within the heated volume, and NP shape does not affect the overall waveform, as shown in Figure 4A,B. The delay observed between photoacoustic pulses (∼0.5 μs) is caused by slight changes in the sample distance from the UST (∼0.749 mm, considering the speed of sound in water [65]).

When dealing with high-powered nanosecond laser pulses, metallic NPs can suffer fragmentation [66]. Moreover, longer AuNRs present higher thermodynamic instability, which leads to lower melting point reshaping [2]. To verify the stability of gold NPs after laser irradiation, the samples were collected in the aftermath of the experiments and subjected to analysis via UV-Vis spectroscopy and SEM (Figure 4C,D). The administered fluence was not sufficient to degrade the NPs.

For the AuNS sample (Figure 5A), the colloidal fluid containing 50 nm nanoparticles produced the highest PA intensity, followed by 5 nm and 100 nm nanoparticles. This observation is consistent with the analysis of the $J_{o}$ values of the nanospheres. This is clearly distinguishable in Figure 5C. In both Figure 5A,C, the highest intensity regions inside the capillary tubes are not centered but instead are slightly shifted towards the right. This is due to the position of the photoacoustic detector at a slight angle from direct light beam incidence (which can be seen in the inset of Figure 2).

The size dependence effect for the AuNR sample on the PA signal is shown in Figure 5B. Here, the distribution of PA intensities is less homogeneous, despite being clearly contained inside the capillary tubes. Moreover, PA (peak) intensities recovered are significantly smaller in Figure 5B than in Figure 5A, which is supported by the lower fluence of the OPO at 808 nm (∼2.7× less than 530 nm) and the random angular orientation of AuNR throughout the sample that effectively reduces the absorbed energy by half. According to Figure 3B, the ${\langle J_{o}\rangle }_{\theta ,\phi }$ of the 90×25 nm nanorod is 42% smaller than that of the 41×10 nm particle. However, this difference is not noticeable experimentally (Figure 5D). The non-uniform character of the PA intensity in the colloids of Figure 5B may be explained by the adhesion of CTAB, a cationic surfactant used to stabilize nanorods [67], to PVC, a polymer with a negatively charged surface [68] that can efficiently adsorb materials possessing positive charges through strong electrostatic interactions [69], thus leading to non-uniform agglomeration of AuNRs on the internal surface of the capillary tube. It is evident from Figure 5B,D that smaller nanorods generate a greater PA signal, consistent with the analysis of ${\langle J_{o}\rangle }_{\theta ,\phi }$ values.

The heating performance of optimized and non-optimized AuNRs were also analyzed through in vivo photothermal therapy procedure in S180-bearing mice. The photoheating of the S180 volume inoculated with 41×10 nm AuNRs led to temperatures of the tumor up to 65.0 °C after 6 min of irradiation, as depicted in Figure 6B. Lower temperatures (about 40.0 °C) at the tumor edges after 10 min of irradiation were observed, and an average tumor temperature of 46.8 ± 0.8 °C (Figure 6A, red line) was recorded, demonstrating the high localization of the therapy. The highly localized hyperthermia generated as a consequence of photothermal conversion may lead to membrane disruption or protein denaturation of targeted cells, resulting in cell death. Likewise, irradiation of S180-bearing mice inoculated by 90×25 nm PEGylated AuNRs samples produced an average temperature increase of 44.6 ± 0.9 °C at the tumor (Figure 6A, blue line). Figure 6C reveals the shorter reach of thermal distribution around the tumor compared to Figure 6B. Consequently, a lower efficacy in therapy is expected for the tissue inoculated with 90×25 nm AuNRs, as the edges of the tumor did not surpass 42.0 °C [14]. Moreover, five minutes of laser irradiation yielded a percentage increase ($100\times \left(T_{41\times 10}-T_{90\times 25}\right)/T_{90\times 25}$) of approximately 9.5% for PTT trials. During in vivo analysis, tumor vascularization promotes heat exchange with blood flow, limiting temperature rise. Additionally, the high inhomogeneity of biological tissues leads to diffuse scattering and irradiation losses.

Laser heating of the tumors inoculated with saline led to temperatures up to 40.7 ± 1.1 °C, as indicated by the black line in Figure 6A. Figure 6A shows that, after 5 min of laser exposure, the S180 volume inoculated with 41×10 nm AuNRs reached thermal equilibrium. This is not the case for the 90×25 nm AuNR inoculation, which requires a longer period of irradiation (more than 10 min) to reach the maximum temperature. Therefore, using optimized nanoparticles reduces the time needed to achieve higher temperatures, thereby decreasing the overall therapy time. Moreover, the 41×10 nm PEGylated AuNRs have also been shown to be efficient nanoheaters for photothermal fungal inactivation [70].

In normal tissues, apoptosis plays a fundamental role in the cell cycle, maintaining homeostasis [71]. In cancer cells, however, apoptosis is inhibited, which enables unregulated proliferation of cancer cells, resulting in tumor growth [13]. Benign tumors undergo expansive growth that leads to smooth borders and sharply demarcate the tumor site from normal tissue. They are typically encased by a fibrous capsule that prevents its spread beyond the layer of tissue in which it developed [72]. In contrast, malignant tumors have an infiltrative character, growing into surrounding healthy tissues and spreading beyond the layer in which they originated [73]. Furthermore, invasive tumors are generally characterized by irregular surfaces [74]. In PTT, apoptosis and necrosis are cell death mechanisms initiated by thermal damage. During necrosis, cell membrane integrity is affected, and damage-associated molecular patterns (DAMPs) released from intracellular content trigger collateral inflammatory responses. During apoptosis, DAMPs are released without affecting membrane integrity and lead to a different pathway through phagocytosis [75]. Nevertheless, membrane integrity is also lost if apoptotic cells are not quickly consumed by phagocytes. This leads to necroptosis [14], a process of secondary necrosis [75].

Particular care must be taken in the use of gold nanorods for bioapplications, as CTAB is a highly cytotoxic surfactant used to aid nanorod synthesis, and which also acts as a surface stabilizer to prevent aggregation [76]. The presence of CTAB in AuNRs directly affects the cytotoxicity of the NP, which can be decreased by the PEGlayering of nanorods. However, PEGlayered nanorods still have CTAB molecules [77], and as the AuNR size increases, its surface area also increases, leading, consequently, to higher NP cytotoxicity due to the presence of larger amounts of CTAB.

Photothermal damage to tumor cells injected with AuNRs was confirmed by histological examination (Figure 7), as the irradiated tissues containing 41×10 and 90×25 nm NPs form solid blocks of coagulative necrosis. The simultaneous presence of apoptosis, necroptosis, and necrosis pathways in both cases are implicated in temperature-dependent cell death patterns. At temperatures below 43 °C (low temperatures), cell killing is ineffective, as cell viability remains above 50%, while at 46 °C (medium temperatures), both apoptosis and necroptosis are the main contributors to cell death [13]. When temperature is raised above 49 °C (high temperatures), cell death become necrosis-dominant [13]. The absence of coagulative necrosis from irradiated tumors inoculated with saline (Figure 7, control) corroborate the low average temperature observed for the control (∼40 °C), below the 43 °C mark, despite the considerably higher temperatures achieved at the center of the tumor. Furthermore, the similar area between necrotic and apoptotic regions of irradiated tumor tissues containing NPs are indicative of medium-temperature PTT. Notwithstanding the respective maximum temperatures of 65.0 °C and 61.8 °C for 41×10 and 90×25 nm, respectively, the average temperatures observed are compatible with medium-temperature PTT.

To minimize necrotic response and maximize beneficial pathways, PTT between the low and medium temperature range (43–46 °C) is preferable. This might be achieved by decreasing laser irradiance or photothermal agent concentration, both of which benefit clinical applications. One such application is externally triggered by on-demand drug delivery systems, in which localized heat is exploited to increase dermal circulation. The increased blood flow improves drug diffusion, enhances therapeutic activity during the sustained release over a defined period of time, and promotes precision dosage control [78].

## 5. Conclusions

A framework for the optimization of metallic nanoparticles for heat generation was established, revealing the size dependence of plasmonic NP optical heating. Here, the Joule number was used as a figure of merit to identify efficient plasmonic nanoheaters. Gold nanospheres with a diameter of 50 nm and nanorods with dimensions of 41×10 nm were identified as effective nanoheaters for visible (530 nm) and infrared (808 nm) excitation. Notably, AuNRs achieve higher average $J_{o}$ values than AuNSs, even considering the possibles orientations of the nanorods. Theoretical results estimate that 41×10 nm AuNRs have an average Joule number of 80, compared to ${\langle J_{o}\rangle }_{\theta ,\phi }$ values of approximately 46 and 9 for 90×25 nm and 135×45 nm samples, respectively.

The photothermal performance of optimal and suboptimal nanostructures was evaluated using photoacoustic microscopy and photothermal therapy procedures, following a temperature increase optimization approach in colloidal gold. For plasmon-mediated PA signal generation, the obtained results indicate that, despite having larger absorption cross sections, the large nanoparticle volume of bigger particles leads to less efficient conversion of light into PA signals. Similarly, 5 minutes of laser irradiation of S180-bearing mice with 41×10 nm produced an approximately 9.5% greater temperature rise than using 90×25 AuNRs in the PTT trials. Although the sizes of optimized nanoparticles were determined, NP toxicity, cellular uptake, NP internalization, surface charge, and targeting remain crucial parameters for in vivo imaging and therapy.

This approach is not limited to AuNSs or AuNRs and can be extended to different shapes and materials. Furthermore, optimizing metallic nanoparticles for heat generation may reduce the concentration of nanoheaters used or decrease light fluence for biosciences applications, paving the way for the development of more economical theranostic agents.

## Data Availability

The data presented in this study are available on request from the corresponding author.

## Abbreviations

The following abbreviations are used in this manuscript:

AuNR	Gold nanorod
AuNS	Gold nanosphere
CTAB	Cetyltrimethylammonium bromide
D	Nanorod diameter
DAMP	Damage-associated molecular pattern
DSO	Digital storage oscilloscope
FEM	Finite element method
FoV	Field of view
IR	Infrared
$J_{o}$	Joule number
L	Nanorod length
LNA	Low-noise amplifier
LSPR	Localized surface plasmon resonance
MPE	Maximum permissible exposure
NIR	Near-infrared
NP	Nanoparticle
OPO	Optical parametric oscillator
PA	Photoacoustic
PAI	Photoacoustic imaging
PAM	Photoacoustic microscopy
PAT	Photoacoustic tomography
PBS	Phosphate-buffered saline
PEG	Polyethylene glycol
PML	Perfectly matched layer
PTT	Photothermal therapy
PVC	Polyvinyl chloride
S180	Sarcoma-180
SEM	Scanning electron microscopy
UFPE	Federal University of Pernambuco
UST	Ultrasonic transducer
UV-Vis	Ultraviolet-visible
